# A Rare Case of Renal Cell Carcinoma With Inferior Vena Cava Invasion: A Life-Threatening Complication

**DOI:** 10.7759/cureus.32978

**Published:** 2022-12-26

**Authors:** Amarachi J Nduji, Zubir S Rentiya, Rowaida Butt, Sanathan Aiyadurai, Albert Annan, Tuba Khan, Syeda Sarah Mahjabeen, Vyapti A Dave, Esther O Apata, Aadil Khan

**Affiliations:** 1 Internal Medicine, Abia State University School Uturu, Uturu Okigwe, NGA; 2 Department of Surgery, MedStar Georgetown University Hospital, Washington, DC., USA; 3 Family Medicine, Avalon University School of Medicine, Mississauga, CAN; 4 Internal Medicine, Caribbean Medical University, Willemstad, CUW; 5 Internal Medicine, Danylo Halytsky Lviv National Medical University, Lviv, UKR; 6 Medicine and Surgery, Ziauddin University, Karachi, PAK; 7 Pathology and Laboratory Medicine, N.T.R. University of Health Science, Hyderabad, IND; 8 Pathology and Laboratory Medicine, Madinah Maternity and Childrens Hospital, Al Madinah Al Munawwarah, SAU; 9 Internal Medicine, Gujarat Medical Education and Research Society (GMERS) Medical College and Hospital, Valsad, IND; 10 Psychiatry, Luhansk State Medical University, Luhansk, UKR; 11 Internal Medicine, Lala Lajpat Rai Hospital, Kanpur, IND

**Keywords:** lung metastases, prosthetic replacement, gerota's fascia, inferior vena cava, renal cell carcinomas (rcc)

## Abstract

Renal cell carcinoma (RCC) arises from the renal tubular epithelial cells and comprises a group of heterogenous renal tumors. Renal tumors can metastasize to involve almost any body organ, the common sites being the lung, liver, bone, brain, adrenal gland, head, neck, and rarely, inferior vena cava (IVC), leading to lethal outcomes. We present a case of RCC with IVC invasion in a patient who presented with right-sided flank pain and gross hematuria. His routine biochemical and hematological parameters were unremarkable, and an abdominal examination revealed a complex renal mass with mild hydronephrosis. The patient underwent contrast-enhanced magnetic resonance angiography with venography, which showed a right renal upper polar mass lesion extending into the right vein obliterating it up to its junction with the IVC. Integrating examination and imaging findings were suggestive of right renal RCC. Our case highlights the importance of standard preoperative MRI imaging to assess IVC invasion and its morphologic features including vessel breach or complete occlusion of the IVC.

## Introduction

Renal cell carcinoma (RCC) can manifest as fatigue, fever, hematuria, backache, hypercalcemia, high blood pressure, or weight loss, constituting 2% of all diagnosed malignancies [1,2]. The etiology of RCC is multifactorial, and smoking and drug exposure are among the most common causes of RCC [2]. RCC invades the inferior vena cava (IVC) and develops a venous tumor thrombus (VTT) in 4-10% of patients [2]. Renal cell carcinoma (RCC) is accompanied by inferior vena cava (IVC) thrombus in up to 10% of the cases, with surgical resection remaining the only curative option. In the case of IVC wall invasion, the operative procedure is more challenging and may even require IVC resection [3]. IVC wall invasion is a poor prognostic indicator, and poorer survival outcomes link to positive renal or caval vein margins [3,4]. The 5-year survival rate was only 26% in patients without resection of the invaded IVC wall; however, it could reach 57% in patients with radical resection [4]. The tumor spread from original sites to nearby structures and distant areas are referred to as metastasis and significantly contributes to morbidity and mortality. A 5- and 10-year disease-free survivals after surgery for T1, T2, T3a, T3b, and T3c tumors are approximately 95% and 91%, 80% and 70%, 66% and 53%, 52%, and 43%, and 43% and 42%, respectively. The presence of distant metastases, including lung and heart, is a tell-tale sign of the primary tumor's aggressiveness. Hematogenous dissemination of tumor cells is the most common route for metastatic lung cancer, with direct venous drainage to the lungs [5]. We report a rare case of RCC with IVC invasion, a rare, life-threatening complication.

## Case presentation

A 60-year-old male with a past medical history of diabetes and hypertension presented with a chief complaint of right-sided flank pain for the past two months. The pain was gradual in onset, progressive, and non-radiating, followed by gross hematuria for the last seventeen days. Associated symptoms included anorexia, nausea, and morning fatigue. He had no history of alcohol abuse. He had a history of smoking one pack/day for ten years and quit smoking twenty years back.

**Figure 1:**
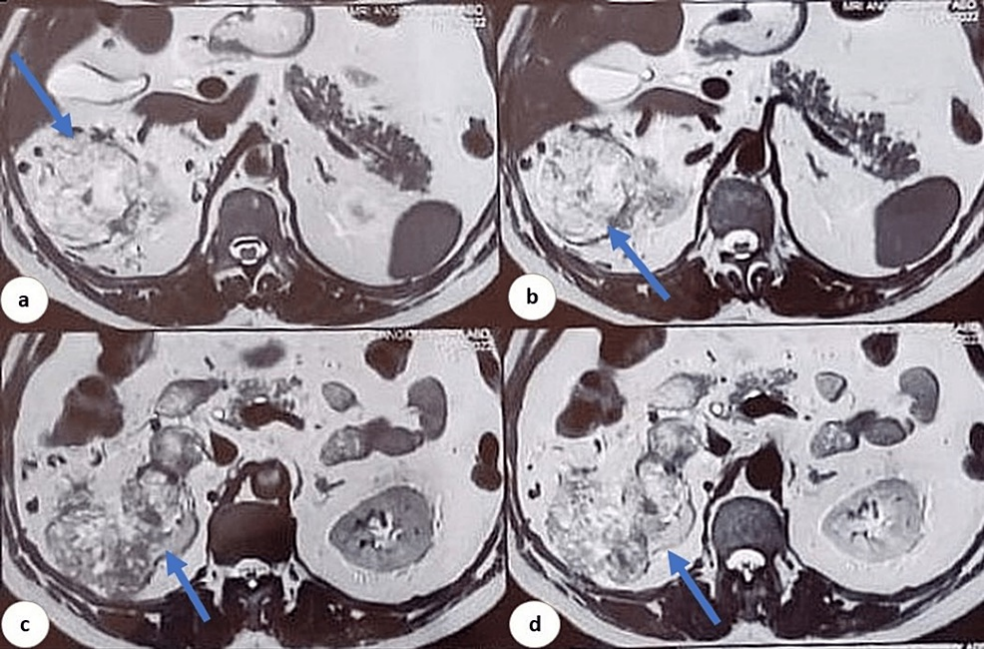
Large heterogeneously enhancing mass lesion involving the upper (a, b) and interpolar region (c, d) of the right kidney.

**Figure 2:**
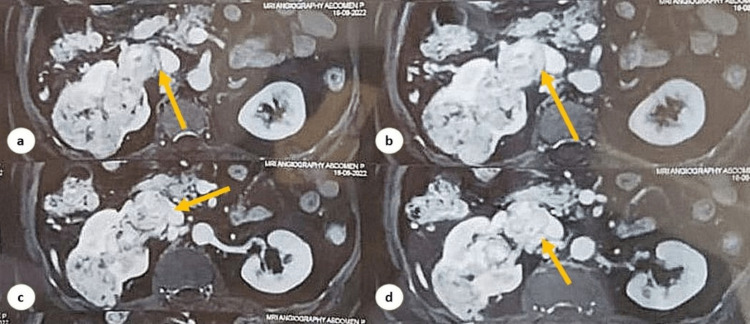
Contrast-enhanced MRA demonstrating right renal upper polar mass lesion obliterating the right vein to the inferior vena cava (a-d). MRA: magnetic resonance angiography.

**Figure 3:**
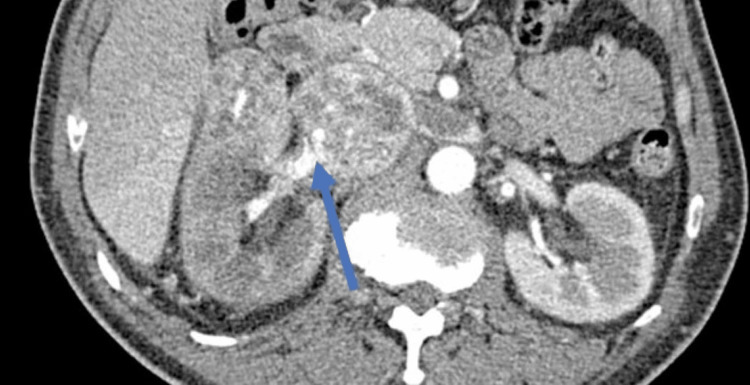
Contrast-enhanced CT showing right renal mass obliterating right renal vein and inferior vena cava.

On examination, he was afebrile and hemodynamically stable. His abdomen was mildly tender, a palpable mass was noted in the right flank region, and the rest of the examination was unremarkable. His routine biochemical and hematological parameters were unremarkable except for anemia (Hb: 7.4 g/dl). Abdominal examination revealed a complex renal mass with mild hydronephrosis. The patient underwent contrast-enhanced magnetic resonance angiography with venography, which showed a right renal upper polar mass lesion extending into the right vein obliterating it up to its junction with the IVC, as shown in Figures 1, 2. The IVC was patent but compressed by mass. Extensive venous collaterals were seen in the perirenal space, and the right renal artery courses were posterior to the anteromedial exophytic component of the neoplasm (Figure 3). Integrating examination and imaging findings were suggestive of right renal RCC.

The patient was managed symptomatically; however, further evaluation with whole-body positron emission tomography (PET-CT) revealed a large heterogeneously enhancing mass lesion involving the upper and interpolar region of the right kidney, having both exophytic and endophytic components involving upper and interpolar calyces, measuring about 7.1 x 10.5 cm in axial dimensions and 7.7 cm in super inferior extent, with contiguous soft tissue lesion extension into right renal vein up to its junction with IVC [maximum standardized uptake value (SUV max): 6.5], with mild heterogeneous uptake noted in adjacent infrarenal IVC. The lesion is seen abutting the right lobe of the liver and involving posterior renal fascia and posterior pararenal space, with right perinephric venous collaterals and fat. Moreover, many tiny avid nodules were noted in the left lung upper lobe and left fissures, highly suggestive of a metastatic lesion.

After further staging, the oncology team advised that the tumor was resectable. Right complete nephrectomy was done after taking proper informed consent. A longitudinal venotomy was done, and the thrombus was dissected from the IVC and extracted quickly. Any remaining residual thrombus was checked. The vena cava cavorraphy was performed with Prolene 4-0 vascular sutures with a double continuous anastomosis technique. Air was also removed before the closure of IVC by tilting the table in the Trendelenburg position and first removing the infrarenal clamp. All perinephric fat and hilar lymph nodes were kept towards the renal side and dissected out with the specimen. The nephrectomy specimen revealed that the tumor was protruding from his renal vein and extended to the superior pole of his kidney. Histopathology of the specimen showed malignant cells with clear cytoplasm and distinct membranes. Cells were uniform, round, irregular, and vesicular nuclei with small nucleoli. The tumor infiltrated the renal parenchyma and extended into the renal capsule, but perirenal fat was not invaded. The tumor extended into his renal pelvis and dilated the renal vein; it adhered to the vessel wall and the inferior vena cava.

The procedure was performed successfully. The patient responded well to treatment and was discharged with outpatient follow-up, and the lesion biopsy confirmed renal cell carcinoma was resected along with bronchoscopy. He was commenced on pazopanib and erythropoietin therapy with a follow-up at six months with CT Scan and then yearly.

## Discussion

RCC is a malignant tumor of the renal tubular cells of the kidneys. The incidence of tumors is, however, less than 10%. Usually, RCC commonly spreads to the lungs, lymph nodes, and liver. We found a rare RCC spreading to the IVC [6]. The prevalence is about 4-10%, in which the tumor invades the IVC and forms a venous thrombus [2]. Gerota's fascia is a layer of connective tissue surrounding the adrenal glands and kidneys. However, the invasion of Gerota's fascia indicates the presence of advanced RCC. Since renal veins drain into IVC, the hematogenous route of the tumor is most likely spread to the lungs through IVC [7]. Rarely, the tumor cells invade the IVC wall forming a venous thrombus, a poor prognostic factor of the disease [6,7].

CT scan is the modality of choice for upper urinary tract carcinomas. CT scan is also used to detect the lung metastasis of advanced RCC. Surgery is the only curative method to excise the entire tumor. The 5-year survival rate is around 50-60%; however, it significantly decreases in the presence of IVC invasion [8]. MRI is more efficient than CT scan in detecting IVC invasion. The management is difficult because of the segmental excision of the tumor and the need for prosthetic replacement to avoid venous insufficiency post-operatively. Anticipating the risk pre-operatively benefits developing the operational plan and prior patient awareness [9].

The Mayo staging system is used to classify the metastatic thrombotic mass in the IVC. Level 1 refers to a thrombotic mass in the IVC at a level less than 2 cm above the renal vein. Level 2 represents the thrombotic mass more than 2 cm above the renal vein. A total of 50% of cases present either as level 1 or level 2. Level 3 indicates the extension of thrombotic mass to the hepatic vein and above, but the mass is below the diaphragm. However, it holds above 40% of the cases. Level 4, the advanced level, indicates the extension of the thrombotic mass above the diaphragm, reaching up to the right atrium. A total of 10% of the RCC cases manifest as level 4. The level measurement is done using preoperative imaging techniques and surgical exploration [10]. 

The only curative approach to date is surgical resection, which has a 5-year survival rate of up to 40-65% for RCC with intravascular growth but is lower in patients with IVC wall invasion [11]. Surgery is more difficult when the IVC wall gets invaded since it might require segmental excision or even prosthetic replacement to prevent postoperative recurrence or venous insufficiency [12]. Typically, the need for prosthetic replacement or segmental resection is determined during surgery. As a result, anticipating IVC invasion before surgery would be a definite benefit for preoperative planning and prior patient knowledge. An essential component of preoperative planning and care is high-quality diagnostic imaging. MRI is more reliable than a CT scan for determining the presence and amount of IVC invasion [12,13].

## Conclusions

RCC with metastases to IVC and lungs leads to a poor prognosis. While CT scans can delineate the renal tumor margins and detect lung metastases, MRI is more efficient in detecting IVC invasion. Mayo staging helps in classifying the thrombotic mass in IVC. Management to date is difficult, as surgical excision is the only curative option. Segmental resection with the need for prosthetic replacement is to be considered, but with limited data available on its use, there is a scope for further studies. Our case highlights the importance of standard preoperative MRI imaging to assess IVC invasion and its morphologic features, including vessel breach or complete occlusion of the IVC.
